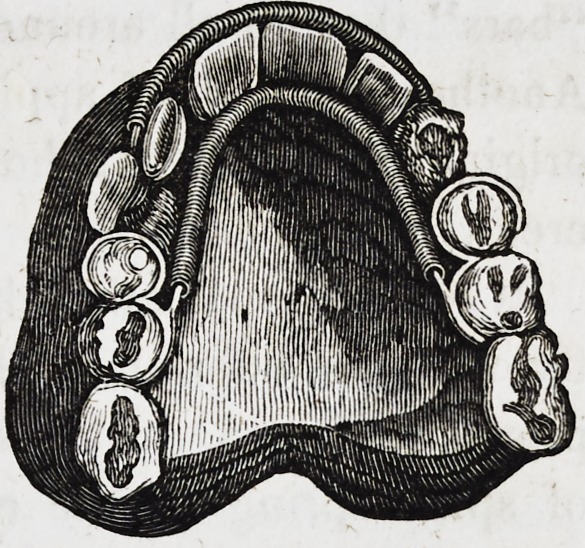# Regulating Teeth by Spiral Springs

**Published:** 1850-10

**Authors:** J. D. White


### ARTICLE XIII.

### *Regulating Teeth by Spiral Springs.*

Messrs. Jones, White & Co.

*Gentlemen:*—Accompanying is an arrangement, with bands and spiral springs, for enlarging the superior maxillary when it is contracted by premature extraction of the deciduous teeth, or any other cause, which I have used with success, in many difficult cases, for many years, as well for expanding the arch as the regulation of teeth. You will perceive that it is calculated to keep up an equal and perpetual pressure on as many teeth as is desirable.

The accompanying model presents an interesting case, and illustrates what are, too often, two inexcusable errors of the past and present generations, and of daily occurrence. 1st. That of extracting the deciduous canine teeth, to give the lateral incisors room, as it is termed, and which, as their unabsorbed fangs leave a deep wound in healing, draws the lateral incisors and first deciduous molars together, contracts the alveolar arch, and allowing the laterals to fall backwards and inwards, dragging the lateral edges of the front incisors along with them, and sometimes before they are long enough to grasp the inferior teeth, in advance, they are much behind them; and 2dly. To mend the matter, when the permanent canine teeth make their appearance, apparently too far outside of the arch, (it is true but a deformed arch,) and only room for half a tooth between the laterals and bicuspids, and in some instances none at all, either they or the first molars, the most valuable teeth in the head, or the bicuspids are doomed to extraction, to make still *more room*, instead of endeavoring to remedy by art what ignorance has done, enlarge the arch and bring out the six or eight stray teeth to the canines, which are in most cases in their proper places. The manner of making it is exceedingly simple: make a plaster model of the mouth in the usual way; then fit a light band around each tooth, joining each other at feather edges between the teeth, instead of filing between, that requires to be thrown outwards. Say, for instance, it is the superior bicuspids of either side, solder those bands together on the palatine sides of the teeth, then solder a short piece of round wire on the same sides of the bands, near their middle, and thick enough to allow of slipping on a spiral spring long enough to extend from one side of the arch to the other, and to lie close along the posterior parts of the front teeth; now if those bands are nicely fitted around the teeth, the apparatus can be worn with comparative comfort; the patient can go to school, (as it is generally in "school days" that the operation is required,) or even into society, without exposing so much gold as those horrible

"bars" that go all around the front part of the teeth and gums. Another method of applying the spiral spring, and which is original with myself, though I do not name it to claim any credit, is as follows:

It often happens that the two lateral incisors are grasped posterior to the inferior teeth, and the front incisors are in their proper positions. By lashing down the extremities of a piece of spiral spring to the necks of the lateral incisors, and the spring extending across the two front incisors, you will perceive that they become a fulcrum for the spring to throw the laterals over the lower teeth. As the spring straightens, the laterals will be brought on a line with the central incisors. It is generally true, that, as the front teeth are harder to force backwards, on account of the alveolar process being thicker on that part than the external alveolar plate, and the teeth being large too, that they afford a sufficient fulcrumage. But if they be not, place a plate in the roof of the mouth, and fasten it to the back teeth, as if it were to set teeth, letting small pieces of plate project forward and downward against the posterior parts of the front teeth, which will prevent the possibility of their being pressed backwards.

But if it be like several cases that I am treating now, where the front teeth are too far out, by the time the laterals are well forward, the fronts will be depressed sufficiently. Or when the median edges of the front teeth present the appearance of bulging out, the spring pressing upon them will level them down properly. If the laterals be too long to sweep over the lower teeth, solder to the plate, if there be one in the mouth, an inclined plane to keep the jaws apart far enough for that purpose. When the plate is not in, lash an inclined plane to one or more of the teeth. Never put a cap over a tooth to receive the direct or full stroke of the jaws in masticating. I have seen cases where the enamel has been literally mashed by that means. As many injuries have been sustained in various ways, in the treatment of irregularity of the teeth, many persons needing attention will not venture to submit to the operation. All cases require close watching, that too much pressure is not produced, so as not to establish periosteal inflammation in teeth that are not fully developed.

I also use the spiral spring for turning teeth that stand across the alveolar ridge. There are a great variety of ways of applying those springs, so as to make a very neat and comfortable apparatus for enlarging or contracting either of the maxillaries, or for operating upon a single tooth.

If the foregoing is worthy a place in your valuable journal, please insert, and oblige

Your humble servant,

*Dental News Letter.*] J. D. WHITE.

## Figures and Tables

**Figure f1:**